# Supplementary material for: Mortality in rheumatoid arthritis patients with pulmonary nontuberculous mycobacterial disease: A retrospective cohort study
Source: PLoS One. 2020 Dec 2;15(12):e0243110. doi: 10.1371/journal.pone.0243110 (PMC7710034; doi:10.1371/journal.pone.0243110)
Supplement: S1 Table — (DOC) [file pone.0243110.s001.doc]

**S1 Table.** Predictive factors for mortality in patients with pulmonary NTM disease (Fine-Gray models using a forced-entry method)

| Predictor variables | All-cause death | | NTM-related death | |
| --- | --- | --- | --- | --- |
|  | Adjusted HRs (95% CIs) * | *p* | Adjusted HRs (95% CIs) * | *p* |
| Age |  |  |  |  |
| ≥80 years | 3.66 (1.55–8.67) | 0.003 | 5.73 (2.17–15.14) | <0.001 |
| 70≤ and <80 | 2.25 (1.05–4.83) | 0.040 | 3.39 (1.24–9.24) | 0.020 |
| <70 years | 1 (reference) | – | 1 (reference) | – |
| Male vs. female | 1.90 (1.02–3.56) | 0.045 | 2.70 (1.40–5.23) | 0.003 |
| Type 2 diabetes (yes vs. no) | 1.47 (0.57–3.75) | 0.42 | – | – |
| Interstitial lung disease (yes vs. no) | 1.74 (0.58–5.27) | 0.33 | – | – |
| Tuberculosis history | 1.91 (0.86–4.26) | 0.11 | 1.34 (0.44–4.10) | 0.60 |
| Serum albumin, g/dl |  |  |  |  |
| <3.0 | 2.60 (0.99–6.85) | 0.054 | 1.58 (0.42–5.91) | 0.50 |
| ≥3.0 and <4.0 | 1.09 (0.57–2.47) | 0.65 | 1.30 (0.53–3.169) | 0.56 |
| ≥4.0 (reference) | 1 (reference) | – | 1 (reference) | – |
| Lymphocyte count, /mm^3^ |  |  | – | – |
| <800 | 2.62 (1.19–5.78) | 0.020 | 2.63 (1.17–5.93) | 0.020 |
| ≥800 and <1000 | 1.21 (0.49–2.99) | 0.69 | 1.49 (0.57–3.90) | 0.42 |
| ≥1000 (reference) | 1 (reference) | – | 1 (reference) | – |
| Causative NTM species |  |  |  |  |
| *M. abscessus* complex | 2.58 (1.00–6.13) | 0.050 | 3.40 (0.98–11.75) | 0.050 |
| *M. intracellulare* | 1.16 (0.59–2.28) | 0.66 | 1.30 (0.55–3.08) | 0.56 |
| *M. avium* | 1 (reference) | – | 1 (reference) | – |
| HRCT pattern of NTM disease |  |  |  |  |
| Cavitary NB/fibrocavitary form | 2.53 (1.28–5.03) | 0.008 | 3.60 (1.48–8.72) | 0.005 |
| Unclassifiable form | 1.25 (0.45–3.47) | 0.67 | 1.12 (0.30–4.17) | 0.87 |
| Non-cavitary NB form (reference) | 1 (reference) | – | 1 (reference) | – |

*Adjusted HRs (95% CIs) are shown for variables that remained in the final Fine-Gray models.

Fine-Gray competing risks analyses were conducted to evaluate the baseline patient characteristics that predict all-cause mortality and NTM-related mortality over time during follow-up. All predictor variables with *p*-values <0.1 in Gray’s test shown in Table 5 (except abnormal HRCT findings) were included in Fine-Gray regression analyses.

RA, rheumatoid arthritis; NTM nontuberculous mycobacteria; HRCT, high-resolution computed tomography; HRs, hazard ratios; CIs, confidence intervals
